# An overview of Triple infection with Hepatitis B, C and D viruses

**DOI:** 10.1186/1743-422X-8-368

**Published:** 2011-07-27

**Authors:** Mehwish Riaz, Muhamad Idrees, Hifza Kanwal, Firoz Kabir

**Affiliations:** 1 National Centre of Excellence in Molecular Biology, University of the Punjab, 87-West Canal Bank Road Thokar Niaz Baig Lahore-53700, Pakistan

**Keywords:** viral hepatitis, HCV, HBV, HDV, liver cirrhosis, hepatocellular carcinoma

## Abstract

Viral hepatitis is one of the major health problems worldwide, particularly in South East Asian countries including Pakistan where hepatitis C virus (HCV) and hepatitis B virus (HBV) infections are highly endemic. Hepatitis delta virus (HDV) is also not uncommon world-wide. HCV, HBV, and HDV share parallel routes of transmission due to which dual or triple viral infection can occur in a proportion of patients at the same time. HBV and HCV are important factors in the development of liver cirrhosis (LC) and hepatocellular carcinoma (HCC). In addition to LC and HCC, chronic HDV infection also plays an important role in liver damage with oncogenic potential.

The current article reviews the available literature about the epidemiology, pathogenesis, transmission, symptoms, diagnosis, replication, disease outcome, treatment and preventive measures of triple hepatitis infection by using key words; epidemiology of triple infection, risk factors, awareness status, treatment and replication cycle in PubMed, PakMediNet, Directory of Open Access Journals (DOAJ) and Google Scholar. Total data from 74 different studies published from 1983 to 2010 on triple hepatitis infections were reviewed and included in this study. The present article briefly describes triple infection with HCV, HBV and HDV.

## Introduction

Hepatitis is a Latin word which means inflammation of liver. At the present time viral hepatitis is a major health problem worldwide, particularly in Asian countries [1]. Hepatitis is caused by different hepatic viruses and it leads to liver related morbidity [2-4]. Mostly hepatic infection is caused by single hepatic virus but sometime infection with multiple viruses may occur and it leads to different management problems. These different problems include higher incidence of morbidity and mortality [5].

As hepatitis C virus (HCV), hepatitis B virus (HBV) and hepatitis D virus (HDV) are transmitted via similar routes that is through blood or blood products so as a result, dual infection and even triple infection can occur in some patients [1,6-10] at the same time. A condition in which all three viruses (Hepatitis B, C and D) occur together in the same patient is called Triple infection [1].

Viral hepatitis is one of the major health issues these days [11,12]. HBV is a partially double stranded, enveloped DNA virus that belongs to the Hepadnaviridae family and Orthohepadnavirus genus. Its size ranges from 40 to 42 nm, replicates in the liver and causes hepatic abnormalities [13,14]. It damages liver through immune mediated mechanisms [15,16]. HBV is the 9^th ^leading cause of death worldwide. There are about 400 million people worldwide who are HCV carriers [17]. It causes cirrhosis, liver failure and hepatocellular carcinoma (HCC) [18]. Annually one million people die due to HBV [19,20]. It has worldwide distribution and is also well documented in Pakistan [21,22].

HCV is a single stranded RNA, enveloped virus that belongs to Flaviviridae family [23]. More than 170 million people are infected with HCV in the world [24]. Like HBV, it replicates in the liver and causes liver abnormalities. Like HBV, it also leads to many serious complications including cirrhosis, liver injury and hepatocellular carcinoma (HCC) [25,26]. These two (HBV and HCV) are the main causes of chronic hepatitis [2,4]. HBV and HCV infection are common in those areas where these two viruses are endemic [27-31].

Hepatitis D virus (HDV) or delta virus is a defective virus. It requires the help of another virus that is hepatitis B virus for its multiplication. It always occur with HBV either in the form of co-infection or super-infection. It is very rare but most severe form of the viral hepatitis [32]. This virus cannot replicate or multiply in the absence of HBV [9]. So we can say that HDV is the result of HBV [33,34]. Worldwide 15 million people positive for hepatitis B are also infected with hepatitis D [35]. Like HBV and HCV, it also plays a significant role in liver abnormalities. It also has oncogenic abnormalities [36,37].

## Literature Search

### Epidemiology of triple viral infection

Cases of triple viral infections and multiple viral infections had been reported from different regions of the world [5,10,38,39]. Mongolia is a country which is highly endemic for triple hepatitis viruses (with HBV, HCV, and HDV). Consequently many cases of triple infection are reported from here. It is also been reported that in Mongolia there is high prevalence of HBV, HCV and HDV [5,38,40-42]. There are also other regions of the world from where triple viral hepatitis infections had been reported including India and China [43,44]. Taiwan is another country which was previously reported as HBV and HCV endemic area but recent studies showed that now it is also endemic for HDV. Many cases of triple infection of hepatitis viruses are also reported from this region. Among the triple hepatitis patients in Taiwan, the prevalence of HBV is 12.6%, HCV is 41.6% and that of HDV is 15.3% [1]. Up to now, there is no report on triple infection caused by hepatitis viruses from Pakistani population [38].

### Pathogenesis

There are many controversies about the role of triple hepatic infection in the pathogenesis of liver disease. It has been reported that triple viral infection does not cause the development of HCC but only one virus that dominate other two viruses in triple viral infection causes this condition of HCC [43].

### Transmission routes

HBV, HCV and HDV viruses are not only biologically different but their life cycles and modes of gene expression are also totally different from each other. Despite of all these differences, they share same routes of transmission [45]. These three viruses (HBV, HCV, and HDV) are the major causes of chronic liver disease. All three viruses (HBV, HCV, and HDV) are transmitted due to direct or indirect exposure to infected blood or body fluids that contain infected blood. Common routes for transmission of these viruses are infected blood transfusion, contaminated syringes, injecting, tattoo and skin piercing with infected instruments, infected household contacts, through infected mother to her baby, by sexual contact with infected person, and via sharing of needles contaminated by infected drug users etc [46]. It is not unusual to find a patient who is co-infected with HBV and HCV or HBV and HDV or with all three viruses (HBV, HCV, and HDV) [1,5-8,47,48].

### Symptoms

Viral hepatitis is of major concern as it leads to morbidity and mortality. Whatever its cause is, all types of viral hepatitis affect liver cells. As a result, many signs and symptoms of various types of hepatitis are almost similar and these are not specific to the viral agent that causes them. Most common symptoms are yellowed eyes or skin (jaundice), fever, fatigue, loss of appetite, joint pain, abdominal pain, diarrhea, nausea, vomiting, dark urine and flu-like symptoms. Some patients may experience few or no symptoms [49-52].

### Diagnosis

Diagnosis of hepatic viral infection is carried out by studying biochemical, virological and histological parameters. Liver function tests (LFT) and serologic assays are also used for detection of antigens and antibodies. As all hepatitis viral infections have same sign and symptoms so it is very difficult to diagnose triple viral infection. A liver function test is available but the pattern of abnormality for acute infection is similar for all types of viral hepatitis. Individually each infection (HBV, HCV and HDV) is confirmed by the presence of the serum surface antigen, hepatitis B, C and D envelope antigen and specific antibodies to the hepatitis B, C and D core. Moreover polymerase chain reaction (PCR) and enzyme linked immunosorbant assay (ELISA) are also used for confirmation [53]. Diagnosis of HCV infection is done by detecting anti-HCV antibody by ELISA method or HCV RNA by PCR method in the serum. HDV is diagnosed by the presence of anti-HDV antibody in the serum using specific ELISA assay. Diagnosis of multiple hepatitis viral infections using one assay is not possible to date. It may be due to low level of awareness among physicians and availability of simple diagnostic tests. But recently scientists used a single integrated protein micro-array for diagnosing multiple viral infections. It can determine two viral antigens (HBsAg, HBeAg) and seven viral antibodies (HBsAb, HBcAb, HBeAb, HCVAb, HDVAb, HEVAb, HGVAb) of human hepatitis viruses in human sera just within 20 minutes [38,54]. However this is very expensive and available in developed countries only.

### Replication status of viruses in triple infection

Although the replication pathway of HBV, HCV and HDV are different but one interesting virological aspect in case of triple infection is that up to what extent each virus affects the replication pathway of other virus and which one is responsible for the pathogenesis of liver disease. The reciprocal influence of HBV, HCV and HDV is still not clear [47,55]. In triple infection with HBV, HCV and HDV, there is interference between the viruses. In such situation one virus might suppresses the effect of others. As some studies have shown that HCV suppresses the effect of HBV and HDV and in some cases it causes sero-clearance of HBV antigens. Similarly there are studies which reported that HBV and HDV suppress HCV effect [48].

Several studies have shown that HDV act as a dominant virus in triple infection and it inhibits the replication of both HBV and HCV [47,48,56-59]. Some studies have also shown that HCV is cleared when patient is super-infected with HBV and HDV [60]. The reason for this could be the inhibition of markers required for their replication [47,58]. Actually large delta antigen of HDV inhibits the host DNA dependent RNA polymerase II which is involved in HBV replication [58,61]. However, some studies reported that HCV act as a dominant virus in triple infection. A study conducted on triple infection of HBV, HCV and HDV by Liaw and colleagues showed that newcomer virus suppress existent viruses [62]. Another study in Australia showed that in patients infected with HBV, HCV and HDV, replication of HBV is suppressed [63]. A study done by Mathurin showed that in Western countries dominant virus is HDV and there is no replication of HCV and HBV in patients with triple infection. Such discrepancies between the studies can be explained as the time of infection and host immune status are different in different parts of the world as a result of which different viruses are dominant in triple infection in different parts of the world [47].

### Disease outcome

As in triple infection all the three viruses are present so scientists are of the view that there are more complications in the early appearance of this disease [64]. A study conducted by Liaw et al on the disease outcome of triple infection with HBV, HCV and HDV reported that this disease is more severe in the acute super-infection stage [62]. Other studies also suggested that infection with HBV, HCV and HDV increases the risk of fulminant hepatitis which is severe impairment of hepatic functions or severe necrosis of hepatocytes [65,66]. Patients with triple infection of HBV, HCV and HDV suffer from more severe liver lesions. They show a high tendency for cirrhosis. They also show increased risk for progression to HCC [63,67-69].

### Treatment

Virological response in case of triple infection varies widely and show many different profiles. As a result of this, level of viruses varies during the therapy and follow-up period. For a proper treatment of triple infection, patient should be followed up yearly and the viral load should be determined which tells us which one virus is gone to be dominant and then we can treat it accordingly [70]. Patients with triple infection involving HBV, HCV and HDV show severe and progressive liver disease. Many studies have shown that when patients with triple infection are treated with interferon therapy, they show resistance to interferon therapy whereas patients having a single viral infection respond to his therapy [48]. Weltman et al., also reported that when seven patients having triple infection were treated with interferon-alpha, six show no response and there is only one patient who normalized serum alanine aminotransferase (ALT) during treatment. So they provide a proof that triple infection show less response to interferon therapy [63].

When patients with triple infection involving HBV, HCV and HDV are treated with lamivudine alone and lamivudine in combination with interferon, they have not shown positive results [71,72]. Another option for patients with triple infection involving HBV, HCV and HDV is liver transplantation. Risk of recurrence of HBV and HCV is decreased in transplanted liver which otherwise may occur due to viral interference phenomenon [73]. Treatment options for triple infection involving HBV, HCV and HDV are very limited. There is no optimal treatment for this up to now [73]. For selecting most appropriate antiviral treatment of triple infection, patient should be monitored carefully and virological assessment should be carried out to determine dominant virus.

### Preventive measures

There are a lot of preventive measures which should be carried out in order to avoid development of triple infection. Some of these are [74]:

1. Screening of donated blood and plasma

2. Routine immunization for infants and high-risk individuals

3. Practicing safe sex

4. Cleaning up blood spills promptly with bleach

5. Avoiding sharing razors, syringes, tooth brushes, nail clippers, or needles, when getting a manicure, a tattoo, or having any body part pierced [74].

## Competing interests

The authors declare that they have no competing interests.

## Authors' contributions

MR searched the literature, organized the data. MR wrote the manuscript. HK and FK helped MR in literature review. MI revised the manuscript. All the authors read and approved the final version of the manuscript.
